# Long-term neuropsychological consequences of severe COVID-19 infection: the NEUROCOG-COVID study

**DOI:** 10.1007/s00415-025-13097-x

**Published:** 2025-04-28

**Authors:** Mylène Meyer, Thérèse Jonveaux, Claire Banasiak, Marine Bié, Leslie Cartz Piver, Anne Chatelain, Céline Dillier, Pascale Gerardin, Coraline Hingray, Christel Jacob, Laura Lavigne, Eloi Magnin, Salomé Puisieux, Louise Tyvaert, Gabriela Hossu, Lucie Hopes

**Affiliations:** 1 Department of Neurology, Hôpital Central, Nancy University Hospital Centre, 29 Avenue du Maréchal de Lattre de Tassigny, 54001 Nancy Cedex, France; 2 Lorraine University, 2LPN, F-54000 Nancy, France; 3 Lorraine University, CIC, Innovation Technologique, Nancy University Hospital Centre, Nancy, France; 4 Department of Neurology, Mercy Regional Hospital Centre, Metz, France; 5 Department of Neurology, Limoges University Hospital Centre, Limoges, France; 6 Lorraine University, IMOPA CNRS UMR 7365, Vandoeuvre-Lès-Nancy, France; 7 CMRR, Centre Neurodéveloppemental Adulte « Hors Normes », Department of Neurology, Besançon University Hospital Centre, 25000 Besançon, France; 8 UMR INSERM 1322, LINC, Laboratoire de Recherches Intégratives en Neurosciences Et Psychologie Cognitive, Franche-Comté University, Besançon, France; 9 Commission of the GREDEVad (Groupe de Réflexion Sur L’évaluation Des Troubles Neurodéveloppementaux de L’adulte) within the GRECO (Groupe de Réflexion Sur L’évaluation Cognitive), Besançon, France; 10 Lorraine University, IADI, INSERM U1254, 54000 Nancy, France

**Keywords:** Cognition, Neuropsychological profile, Executive disorder, Cognitive complaint, COVID-19

## Abstract

**Background:**

Recent studies have confirmed the presence of cognitive disorders, which may be maintained over the long term and associated with psychological disorders following COVID-19 infection. The aim of our study was to characterize long-term cognitive and psychiatric disorders in patients younger than 65 years hospitalized for severe COVID-19 infection.

**Methods:**

All patients who were hospitalized between October 2020 and July 2021 for severe COVID-19 infection with a cognitive complaint according to the QPC questionnaire were selected. They underwent a systematic neuropsychological evaluation assessing cognitive functions, psychological processes, and quality of life (QOL).

**Results:**

The QPC was offered to 293 patients, 129 of whom had a cognitive complaint. A total of 74 (57% men) of these patients, aged approximately 55 years, had undergone a full neuropsychological evaluation 337.38 ± 25.11 days after hospital discharge. Seventy-three percent presented with cognitive disorders, including executive disorders (66%), memory disorders (31%), language disorders (19%), and other instrumental disorders (12%). Single-domain impairment was found in 54% of patients, with predominantly “dysexecutive syndrome” (83%) profile. There was no difference between the groups concerning psychological impairment. Patients with a “dysexecutive syndrome” profile reported poorer mental QOL than did the other patients (*p* < .05).

**Conclusions:**

Cognitive disorders are common after severe COVID-19. The consideration of these factors is essential in the management of patients with long-term COVID-19, especially considering their impact on patients' QOL. Comprehensive neuropsychological assessment helps to identify the factors contributing to cognitive complaints to optimize multidisciplinary management, particularly when not related to cognitive disorders on testing.

## Background

As early as 2020, numerous international scientific articles reported the possible presence of cognitive disorders in patients infected with the SARS-CoV-2 virus [1, 2]. The onset of cognitive complaints is said to be very frequent in the aftermath of COVID-19 infection, particularly in patients who have experienced severe infection. In general, “brain fog” is often described as slowed thinking, difficulties with focused attention, forgetfulness, and a subjective sense of confusion [3]. Most often, the presence of a cognitive complaint is associated with the presence of cognitive disorders on neuropsychological assessment [3, 4], particularly executive dysfunction [5]. However, cognitive complaints do not correlate with the severity of objective cognitive impairment on neuropsychological assessment [6], and can persist over time [3, 4, 7, 8].

While some articles testify to the absence of neuropsychological consequences of the SARS-CoV-2 virus [9], a growing number of studies seem to validate a causal effect of COVID-19 on the central nervous system, possibly manifesting as neuropsychiatric disorders, both in the acute and long-term phases [1, 5, 6, 10, 11]. Studies seem to consistently show that the development of neuropsychological disorders after COVID-19 infection is independent of the severity of the infection, i.e., mild infection requiring no respiratory assistance and hospitalization versus severe infection with respiratory distress requiring intensive care [10–12]. Although cognitive impairment may be more pronounced in patients with severe infections [11, 13], it also seems possible in asymptomatic patients [5]. The long-term course of cognitive impairment is again variable; while it tends to persist after onset, it may increase over time, particularly in patients with moderate or severe infection, or, conversely, gradually improves in patients with mild infection [14]. As in other neurological pathologies [15], functional cognitive disorders could be present in association with the organic consequences of COVID-19 infection.

The various exhaustive neuropsychological assessments proposed seem to testify to a high prevalence of executive and attentional impairment in patients with COVID-19 infection [1, 5, 10, 16, 17] and, more rarely, of deficits in episodic memory, language, or visuoconstructive impairment. It also appears that executive deficits are significantly correlated with attentional deficits [5], either because of shared fronto-subcortical circuits or because of a causal link between attentional deficits and their repercussions on executive functioning.

These cognitive disorders often appear to be associated with psychiatric disorders, particularly anxiety and depressive disorders, as well as posttraumatic stress disorders [8, 18, 19], manic disorders, apathy, dissociative disorders, insomnia, somnolence, or pathological fatigue [6]. While psychiatric symptoms alone may not be the cause of cognitive impairment in post-COVID-19 patients, they are known to increase cognitive impairment [10]. Nevertheless, depression [3] may be a risk factor for the development of long-term cognitive disorders after COVID-19 infection.

However, through the abundant literature, significant methodological limitations have been identified in studies about neuropsychological disorders [20]. Indeed, some studies have speculated on the consequences of the virus on the brain, and therefore on neuropsychiatric consequences in patients, on the basis of transpositions of previous experiences with the SARS-CoV and MERS viruses [21, 22]. Other studies have identified cognitive disorders on the basis of limited neuropsychological assessments or even screening tools [16, 23, 24, 26]. There is also great variability in terms of the severity of infection criteria (hospitalized versus nonhospitalized patients) or the time of assessment [acute phase [24, 26, 27], postacute phase-1 month [25, 28–30], 4–6 months posthospitalization [7, 31, 32], and 1-year postcovid hospitalization [3, 13]].

The aim of our study was to characterize the neuropsychological profile of patients who have had severe COVID-19 infection by means of an extensive neuropsychological assessment at 9 months from hospital discharge. This assessment was proposed for a relatively young population to limit the bias of age-related comorbidities in patients clearly impacted by the repercussions of cognitive disorders on daily life.

## Methods

### Design

NEUROCOG-COVID is a longitudinal prospective, multicenter, nonrandomized, and open study (Clinical Research Program 2021-A00447-34) approved by the ethical committee and registered on the ClinicalTrials.gov website (NCT04937582).

The inclusion criteria consisted of being aged between 18 and 65 years and having been hospitalized for more than 72 hours between October 2020 and July 2021 for a defined COVID-19 infection (positive RT-PCR test and/or typical chest scanner images). Patients had to be affiliated with social security regimens. Patients needed to have sufficient French language understanding to perform neuropsychological assessments. Patients were recruited from 4 French university hospitals (Nancy, Besançon, Metz, Limoges), and they provided written informed consent to participate in the study.

The exclusion criteria consisted of chronic neurological disorders (neurodegenerative disorders, epilepsy, and multiple sclerosis) and/or major cognitive disorders (stroke, brain injury) and/or chronic psychiatric disorders (bipolar or psychotic disorders) prior to COVID-19 infection. Patients with a history of addiction or comorbidities with post-COVID-19 consequent cognitive disorders were excluded, as were patients who were unable to perform neuropsychological assessments due to extensive post-COVID-19 cerebral lesions or moving difficulties.

**Fig. 1:**
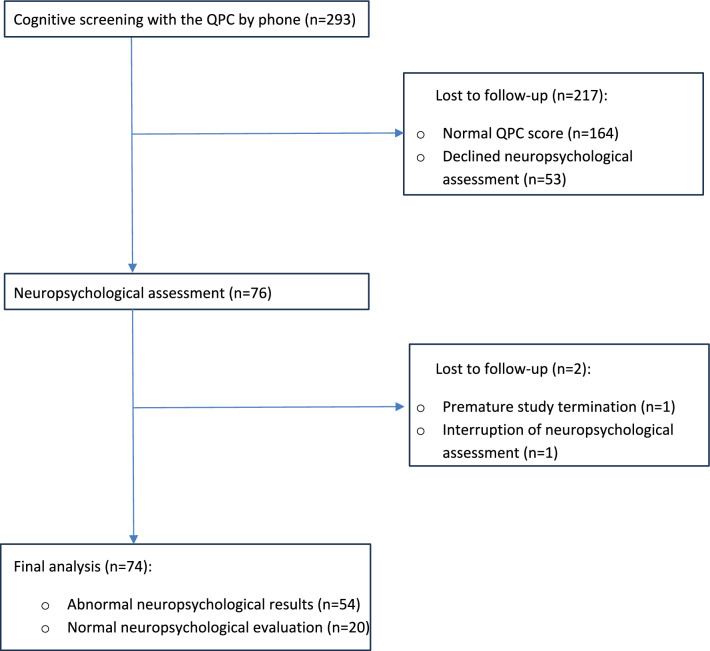
CONSORT flow diagram of screening and inclusion of patients for the NEUROCOG-COVID-19 study

The medical files of patients hospitalized for severe COVID-19 infection between October 2020 and July 2021 (Fig. 1) corresponding to the inclusion criteria were extracted. Each patient on this list was systematically called by phone to perform a cognitive complaint screening based on the QPC (Questionnaire de Plainte Cognitive [33]), which is a 10-item questionnaire with yes‒no responses, with a general score ≥3 corresponding to significant cognitive complaints.

#### Neuropsychological assessment

Patients with post-COVID-19 cognitive complaints (QPC ≥3) were proposed to participate in the NEUROCOG-COVID-19 study and to perform an extensive neuropsychological assessment with a trained neuropsychologist. This evaluation consists of an assessment of cognitive functions, for which the tests were chosen to explore attentional and executive capacities, while being relatively adapted to their age and professional condition. The cognitive battery is composed of the MOCA scale [34], the RLS-15 [35], the Rey‒Osterrieth complex figure (ROCF, [36]), the coding task and the digit span from the WAIS-IV [37], tasks of the TAP battery (working memory, flexibility, and incompatibility; [38]), the verbal fluency task [39] and the shopping task [40], the denomination task DO80 [41], and the brief Mahieux-Laurent-apraxia scale [42].

This assessment was supplemented by an evaluation of several psychological dimensions, assisted by a neuropsychologist. Patients were evaluated for depression [MADRS [43]], anxiety [Hamilton Anxiety Rating Scale (HARS, [44]), apathy (LARS, [45]], psychotrauma [PCL-5, [46], and dissociation (DES, [47]]. Additional questionnaires have been proposed to investigate quality of life [QOL; SF-36, [48]], pain [10-point visual-analogical scale, DN4 scale [49]], tiredness [Chalder scale [50], and somnolence (Epworth scale [51]].

The detailed neuropsychological tests and psychological questionnaires used are presented in the Appendix.

The presence of a neuropsychological disorder, i.e., performance in a cognitive domain outside the expected norms for age and socioeducational level, was characterized by the neuropsychologist by the predominance of at least one of these cognitive impairments: dysexecutive syndrome (with failure to perform at least two tests), memory impairment (with failure in episodic memory performances), language impairment (with failure to perform language tests),and another instrumental profile (with failure to perform visuoconstructive and/or agnosia and/or apraxia tests). Then, with the help of these neuropsychological data, the neuropsychologist classified each neuropsychological profile according to whether it appeared as a “dysexecutive syndrome” profile, a “memory impairment” profile, a “no predominance profile” (i.e., more diffuse cognitive abnormalities not being classified in the previous categories), or a “normal cognition” profile for patients with normal neuropsychological evaluations, not meeting the criteria for previously described impairments. We sought to determine the factors contributing to cognitive complaints, the presence of neuropsychological impairment at assessment, and the presence of dysexecutive syndrome in our sample.

### Statistical analysis

Normally distributed continuous variables are expressed as the means +/− standard deviations, nonnormally distributed data are expressed as the medians and interquartile ranges (median [Q1,Q3]), and categorical variables are expressed as the frequencies (percentages). The normality of the distribution was assessed using the Shapiro‒Wilk test. The number of missing values for all measurements was reported.

A statistical description of patients’ characteristics according to neuropsychological group was performed via the Chi-square test if assumptions were satisfied or with Fisher’s exact test for categorical data. For continuous data, Student’s t test was used if the sample size or normality assumption was met; otherwise, the Wilcoxon test was used. Tests were also adjusted for multiple comparisons according to the Bonferroni–Holm correction when appropriate.

Multivariate logistic regression analysis was conducted to identify factors associated with the presence of cognitive complaints, neuropsychological impairment, and dysexecutive syndrome. Multivariate regression analysis was conducted to identify factors associated with neuropsychological impairment. Relevant factors were identified after univariate analysis with a p value < 15% and were included in the model.

Statistical analyses were conducted using R 4.3.1 (R Core Team (2024). R: A Language and Environment for Statistical Computing. R Foundation for Statistical Computing, Vienna, Austria. <https://www.R-project.org/>.), including its *glmulti* library (https://www.jstatsoft.org/article/view/v034i12). The statistical significance level was set to *P *< 0.05.

## Results

### Sample

Two hundred and ninety-three patients were contacted by phone; 164 had a normal score on the QPC questionnaire and were excluded. Among the 129 patients who had significant cognitive complaints on the QPC, 76 agreed to complete the neuropsychological assessment. These patients were included in the study (Fig. 1) 337.38 +/− 25.11 days after hospital discharge for COVID-19 infection. Two patients did not complete the assessment and were ultimately excluded.

The sample consisted of 74 patients (57% men) who had undergone a full neuropsychological evaluation 27.5 [17.25–42.75] days after the phone call for the QPC. They were aged approximately 55 years [50–59.75]. Patients had a junior (34%, *n* = 25) or senior (32%, *n* = 24) high school education, and 32% (*n* = 24) had a postgraduate degree. The scholar level failed for one patient. The complete sample data are presented in Table 1.

**Table 1:** Demographic data of the sample (*N* = 74)

	Med(Q1-Q3)	*P*
Age	55(50–59.75)	0.34
	*N *(%)	
Men	32 (43)	*P*
Women	42 (57)	0.25
School degree		
Junior high school education	25 (34)	0.25
Senior high school education	24 (32)	
Postgraduate degree	24 (32)	
NA	1 (1)	

### Cognitive profiles

The detailed neuropsychological profile is shown in Table 2 and Table 3.

**Table 2:** Descriptive data concerning the type of neuropsychological impairment present in the total sample (*N*=74) and per subgroup

	Total sample *N* = 74	Normal cognition (*n* = 20)	Memory impairment (*n* = 8)	No predominance (*n* = 7)	Dysexecutive syndrome (*n* = 39)	P
Type of impairment, n (%)						
No impairment	20 (27)	20 (100)	0 (0)	0 (0)	0 (0)	0.0005**
Single domain	29 (39)	0 (0)	5 (62)	0 (0)	24 (62)	
Multiple domain	25 (34)	0 (0)	3 (38)	7 (100)	15 (38)	
Executive impairment						
Absent	5 (7)	0 (0)	5 (62)	0 (0)	0 (0)	0.0005**
Present	49 (66)	0 (0)	3 (38)	7 (100)	39 (100)	
Memory impairment						
Absent	31 (42)	0 (0)	0 (0)	0 (0)	31 (79)	0.0005**
Present	23 (31)	0 (0)	8 (100)	7 (100)	8 (21)	
Language impairment						
Absent	40 (54)	0 (0)	8 (100)	2 (29)	30 (77)	0.0005**
Present	14 (19)	0 (0)	0 (0)	5 (71)	9 (23)	
Other instrumental impairment						
Absent	45 (61)	0 (0)	6 (75)	5 (71)	34 (87)	0.0005**
Present	9 (12)	0 (0)	2 (25)	2 (29)	5 (13)	

^*^Identifies significant differences with *p* < 0.05

^**^Identifies significant differences with *p* < 0.001

**Table 3:** Neuropsychological performance of the total sample (*N*=74) and each subgroup

Tests scores (med, Q1, Q3 or med/SD)	Total sample, *N* = 74	Normal cognition (*n* = 20)	Memory impairment (*n* = 8)	No predominance (*n* = 7)	Dysexecutive syndrome (*n* = 39)	P	P bonferroni correction
MOCA	27 (25–29)	**29** (29–30)^a,b^	27 (25.75, 28.5)	**25** (24.5, 27.5)^a^	**27** (25, 28)^b^	0.00043	^a^0.011* ^b^0.001**
RLS-15 MR	10.3 (9.43, 11)	**11.1** (10.3, 11.95)^a,b,c^	**9** (8.47, 9.9)^a^	**9.6** (7.9, 9.85)^b^	**10.3** (9.4, 10.8)^c^	9,00E-05	^a^0.002* ^b^0.009* ^c^0.027*
RLS-15 LC	55.84 ± 16.91	**66.95** ± 16.4^a,b^	**42.41** ± 11.98^a^	**39.96** ± 14.8^b^	55.75 ± 14.29	5,00E-05	^a^0.005* ^b^0.009*
RLS-15 DR	12 (10, 13)	**12.5** (12, 15)^a,b^	**10** (8, 11.25)^a^	**9** (7.5, 10.5)^b^	12 (10, 13)	0.00037	^a^0.012* ^b^0.003*
ROCF IR	20 (17, 23.75)	**22.5** (20, 26)^a^	15 (11.25, 23.5)	**14** (11.5, 19)^a^	20 (17.5, 23)	0.01816	^a^0.042*
ROCF DR	20 (16, 23)	**23** (18.75, 24.5)^a^	15.5 (11.25, 22.25)	**15** (13, 19)^a^	20 (17.5, 23)	0.02136	^a^0.046*
ROCF copy	34 (32, 35.75)	35 (34, 36)	33 (31, 33.5)	32 (30.5, 33)	34 (32, 36)	0.03133	**ns**
Code WAIS-IV	59.55 ± 18.89	**69.8** ± 10.28^a^	61.62 ± 12.63	60.29 ± 35.25	**53.74** ± 17.78^a^	0.01832	^a^0.002*
TAP Flexibility RT med	902 (722, 1076)	**718** (552.25, 881.25)^a,b^	**681.5** (661, 778.5)^c^	**1120** (941, 1356.75)^a^	**1012** (850, 1255)^b,c^	1,00E-04	^a^0.020* ^b^0.001** ^c^0.043*
TAP Flexibility RT SD	257 (203, 368)	**188.5** (137, 218.75)^a, b^	200.5 (190.25, 280.75)	**398.5** (273.5, 558.75)^a^	**327** (244, 436.5)^b^	< 1e-05	^a^0.016* ^b^<0.001**
TAP Flexibility CA	96 (89, 98)	**98** (96, 100)^a^	98 (96, 98.5)	77 (67.25, 94.25)	**94** (85, 98)^a^	0.00317	^a^0.009*
TAP Flexibility Errors	2 (1, 6)	**1** (0, 2)^a^	1 (0.75, 2)	11.5 (2.75, 16.5)	**3** (1, 7.5)^a^	0.00432	^a^0.014*
TAP Incompatibility RT med	541 (487.75, 590)	**493.5** (449.5, 529.25)^a^	455.5 (419.75, 522.5)	550.5 (538, 566)	**559** (535.5, 617.75)^a^	0.00113	^a^0.002*
TAP Incompatibility RT SD	112.5 (83.75, 172.5)	**81.5** (72.75, 102.75)^a^	**83.5** (72, 112.5)^b^	139 (97, 180.25)	**147** (114.25, 191)^a, b^	< 1e-05	^a^<0.001** ^b^0.052
TAP Incompatibility CA	58 (54.75, 59)	**59** (59, 60)^a^	58 (49, 59)	58.5 (57.25, 59)	**56** (51.25, 59)^a^	0.00181	^a^0.001*
TAP Incompatibility Errors	1 (1, 4)	**1** (0, 1)^a^	1.5 (1, 2.5)	1 (1, 2.5)	**2.5 (**1, 7)^a^	0.00593	^a^0.005*
TAP Working memory RT med	644.08 ± 171.31	**561.3** ± 131.58^a^	592.5 ± 82.51	627.67 ± 110	**713.97** ± 194.07^a^	0.01097	^a^0.003*
TAP Working memory omissions	3 (1, 5)	**0.5** (0, 2.25)^a^	2.5 (1, 3.25)	4 (3, 8)	**3** (2, 6)^a^	0.00524	^a^0.008*
TAP Working memory errors	3 (1, 6)	**2** (0, 2.25)^a^	3 (2.5, 5.75)	3 (1.5, 4.5)	**6** (2.5, 7.5)^a^	0.00585	^a^0.005*
Literal fluency	21.31 ± 7.22	**27.55** ± 5.58^a^	22.25 ± 5.55	20.14 ± 6.84	**18.13** ± 6.33^a^	< 1e-05	^a^<0.001**
Categorical fluency	31.68 ± 8.76	**37.25** ± 8.48^a^	31.12 ± 4.52	27.71 ± 10.31	**29.64** ± 8.21^a^	0.00652	^a^0.008*
ROCF copy duration	172.5 (127, 214)	144 (122.75, 210)	134 (103, 155.75)	183 (145.5, 202)	190 (135.5, 244)	ns	
TAP Working memory RT SD	220.94 Â± 92.14	190.85 Â± 91.71	221.38 Â± 83.28	189.67 Â± 98.01	246.29 Â± 90.01	ns	
Shopping task duration	6.08 (4.44, 8.03)	6.2 (5.36, 7.48)	4 (3.18, 7.25)	4.7 (4.4, 5.22)	6.18 (4.5, 9.21)	ns	

^*^Identifies significant differences with *p* < 0.05

^**^Identifies significant differences with *p* < 0.001

Bold values to highlight their statistical significance

Although all included patients presented with an initial cognitive complaint measured by the QPC one year after their hospitalization, some of them (*n* = 20) presented a “normal cognition” profile. These patients performed within the expected norms on the proposed neuropsychological tests and did not meet the criteria for cognitive impairment as defined in our study.

The other patients (*n *= 54) presented abnormal cognitive evaluations. Fifty-four percent (*n* = 29) of them had single-domain impairment and were assigned to the “dysexecutive syndrome” group (83%, *n* = 24) and, to a lesser extent, to the “memory impairment” group (17%, *n* = 5). The remaining patients (46%, *n* = 25) had multidomain impairment, the majority of whom belonged to the “dysexecutive syndrome” group (60%, *n* = 15) and a few to the “no predominance” group (28%, *n* = 7) or the “memory impairment” group (12%, *n* = 3).

The “dysexecutive syndrome” group performed less well than the “normal cognition” group did in the coding task (53.74 +/− 17.78 vs. 69.8 +/− 10.28, *p* <.01) and provided fewer words in the literal task (18.13 +/− 6.33 vs. 27.55 +/− 5.58; *p* <.001) and the categorical task (29.64 +/− 8.21 vs. 37.25 +/− 8.48; *p* <.01). Compared with “normal cognition” patients, “dysexecutive syndrome” patients presented slower reaction times (1012 ms [850–1255] vs. 718 ms [552.25–881.25], *p* < 0.001), fewer good answers (94 [85–98] vs. 98 [96–100], *p* < 0.01), and more errors (3 [1–7.5] vs. 1 [0–2], *p *< 0.05) during the flexibility task. The same was observed for the incompatibility task, with the “dysexecutive syndrome” patients presenting slower reaction times (585 ms [535.5–617.75] vs. 493.5 ms [449.5–529.25], *p* < 0.01), fewer good answers (56 [51.25–59] vs. 59 [59–60], *p* < 0.01), and more errors (2.5 [1–7] vs. 1 [0–1], *p* < 0.01) than did the “normal cognition” patients. During the working memory task, the “dysexecutive syndrome” patients also presented slower reaction times (713.97 ms +/− 194.07 vs. 561.3 ms +/− 131.58, p < 0.01), more omissions (3 [2–6] vs. 0.5 [0–2.25], *p* < 0.01), and more errors (6 [2.5–7.5] vs. 2 [0–2.25], *p* < 0.01) than did the “normal cognition” patients. The “dysexecutive syndrome” patients appeared slower than the “memory impairment” patients (1012 ms [850–1255] vs. 681.5 ms [661–778.5], *p* < 0.05) during this flexibility task.

Patients in the “dysexecutive syndrome” (27 [25–28], *p* < 0.001) or “no predominance” (25 [24.5–27.5], *p* < 0.05) groups had lower scores on the MOCA test than did “normal cognition” patients (29 [29, 30]).

The “no predominance” patients appeared slower than the “normal cognition” patients did during this flexibility task (1120 ms [941–1356.75] vs. 718 ms [552.25–881.25], *p* < 0.05). With respect to nonverbal episodic memory, spontaneous retrieval performance was significantly lower in immediate (“no predominance”: 14 [11.5–19] vs. “normal cognition”: 22.5 [20–26], *p *< 0.05) and delayed recall (“no predominance”: 15 [13–19] vs. “normal cognition”: 23 [18.75–24.5], *p* < 0.05) for patients in the"no predominance"group than for those in the “normal cognition” group.

With respect to verbal episodic memory, the mean retrieval capacity during the learning phase of the RLS-15 was significantly lower for patients in the"no predominance"(9.6 [7.9–9.85], *p* < 0.01),"dysexecutive syndrome"(10.3 [9.4–10.8], *p* < 0.05), and"memory impairment"(9 [8.47–9.9], *p* < 0.01) groups than for patients in the normal (11.1 [10.3–11.95]) neuropsychological workup. The learning index and delayed recall scores were significantly lower for patients in the"no predominance"(learning index: 39.96+/− 14.8, *p* < 0.01; delayed recall: 9 [7.5–10.5], *p* < 0.01) and"memory impairment"(learning index: 42.41+/− 11.98, *p* < 0.01; delayed recall: 10 [8–11.25], *p* < 0.05) groups than for those with a normal (learning index: 66.95+/− 16.4; delayed recall: 12.5 [12–15]) assessment.

No other significant differences between groups were observed concerning episodic memory or executive and instrumental functions.

### Mood, psychological dimensions, and quality of life

The results of the psychological, psychiatric, and QOL scales are shown in Table 4.

**Table 4:** Psychological, psychiatric, and quality-of-life scores for the total sample (*N* = 74) and for each subgroup

Tests scores (med, Q1, Q3 or med/SD)	Total sample *N* = 74	Normal cognition (*n* = 20)	Memory impairment (*n* = 8)	No predominance (*n* = 7)	Dysexecutive syndrome (*n* = 39)	P	P bonferroni correction
MADRS	7 (4.25, 15)	6.5 (4, 10.25)	5 (4.5, 9)	5 (3, 14)	10 (5, 18.5)	ns	
HARS	10 (6, 21.75)	9.5 (5.75, 13.75)	5.5 (4.75, 7.25)	11 (9.5, 24.5)	15 (7, 24.5)	ns	
LARS	− 29 (− 33, − 21)	− 28 (− 34, −22.75)	− 29.5 (− 32, − 27)	− 31 (− 32.5, − 19)	− 28.5 (− 32.75, − 18)	ns	
PCL-5	12 (7.5, 28.5)	10.5 (5, 17.25)	11.5 (9.5, 13.25)	18 (11, 19)	16 (8, 36)	ns	
DES total	6.64 (3.92, 15.71)	6.24 (4.08, 9.99)	5.36 (3.81, 8.39)	6.61 (5, 10.4)	8.75 (3.39, 17.5)	ns	
DN-4	2 (0, 5)	1 (0, 2.5)	0.5 (0, 5.25)	5 (1, 6.75)	2 (0, 5)	ns	
Chalder	18.44 ± 6.3	17.95 ± 6.88	17 ± 6.55	20.86 ± 5.43	18.56 ± 6.21	ns	
Epworth	8 (4.5, 11)	7 (4, 11.25)	6 (5, 7.75)	9 (7.5, 10)	9 (4.75, 12)	ns	
SF-36 physical	52.3 (32.16, 77.35)	67.81 (47.13, 80.78)	76 (73.79, 84.16)	52.92 (28, 64.06)	45.11 (20.73, 69.84)	0.04554	ns
SF-36 mental	61.35 (34.58, 77.42)	**72.12** (49.89, 78.35)^a^	**80.61** (69.83, 82.47)^b^	43.46 (27.56, 62.52)	**46.45** (21.82, 67.45)^a, b^	0.00463	^a^0.049* ^b^0.048*

^*^Identifies significant differences with *p* < 0.05

^**^Identifies significant differences with *p* < 0.001

Bold values to highlight their statistical significance

No significant differences were detected between the four groups or between patients with and without abnormal neuropsychological performance in the dimensions of depression (MADRS), anxiety (HARS), apathy (LARS), psychotrauma (PCL-5), dissociation symptoms (DES), neuropathic pain (DN-4), fatigue (Chalder), or somnolence (Epworth).

No significant differences were observed for these variables when patients with and without cognitive disorders were compared (data presented in the appendix, Table 9). However, when focusing on scale scores, some patients had scores that reflects a particular psychiatric symptomatology. In fact, 51% of our sample showed depressive symptoms according to the MADRS, mainly indicating mild depression (32%), to a lesser extent moderate depression (16%) and very rarely severe depression (3%). More than half of the patients in the sample (78%) had anxiety according to the HARS, mainly mild anxiety (39%) and, less frequently, major anxiety (39%).

While most of the samples did not show apathy according to the LARS, 14% of the samples tended toward apathy. Some others reported moderate (5%) or severe (7%) apathy, and these patients were in the group with cognitive disorders.

Moreover, 12% of patients had an abnormal score on the DES dissociation scale, the majority of whom belonged to the group with cognitive disorders (15%) compared to patients without cognitive disorders (5%). Concerning psychotrauma, 20% of the sample presented scores indicating PTSD on the PCL-5; 15% of the patients with PTSD were from the group with no cognitive disorders, and 22% were from the group with cognitive disorders.

Thirty-four percent of patients had neuropathic pain (DN4), mainly in the cognitive impairment group (41%), compared with the noncognitive impairment group (15%).

Approximately 65% of the total sample had pathological fatigue (Chalder), with no difference according to the presence or absence of cognitive disorders. Eight percent of the patients presented with signs of excessive drowsiness, whereas 38% of the patients presented with sleep deficit, again with no differentiation according to the presence or absence of cognitive disorders.

Mental QOL (SF36) was lower in patients with cognitive disorders (47.83 (26.88, 75.25)) than in patients with no cognitive disorders (72.12 (49.89, 78.35); *p* = 0.039). The mental aspects of QOL were significantly lower for the “dysexecutive syndrome” group (46.45 [21.82–67.45]) than for the “memory impairment” (80.61 [69.83; *p* < 0.05]) or “normal cognition” (72.12 [49.89–78.35; *p* < 0.05]) groups.

No significant difference between groups was observed for the physical dimension of QOL (SF-36).

### Demographic, cognitive, and psychiatric interactions

Data concerning regression analysis over cognitive complaints, neuropsychological impairment, and dysexecutive profiles are presented in Table 5, Table 6, and Table 7, respectively.

**Table 5:** Neuropsychological and demographic factors contributing to the presence of a cognitive complaint in post-COVID-19 patients

Cognitive complaint (QPC score)				
Coefficients				
	Estimate	Std. error	t value	Pr(>|t|)
(Intercept)	2.980152	2.868446	1.039	0.3033
MOCA score	−0.047744	0.092009	−0.519	0.6059
Flexibility task (RT)	0.004508	0.001896	2.377	0.0209 *
Fatigue scale score (Chalder)	0.098802	0.039873	2.478	0.0163 *
Neuropathic pain (DN-4)	0.226259	0.095773	2.362	0.0217 *
Dissociative score (DES)	0.013459	0.018312	0.735	0.4654

^*^Identifies significant differences with *p* < 0.05

^**^Identifies significant differences with *p* < 0.001

**Table 6:** Neuropsychological and demographic factors contributing to the presence of neuropsychological disorders in post-COVID-19 patients

Neuropsychological disorder							
Coefficients							
	Estimate	Std. Error	z value	Pr(>|z|)	OR	2.5%	97.5%
(Intercept)	28.50182	12.67569	2.249	0.0245*	2.388815e+12	33520.37	5.172296e+33
MOCA score	− 0.49818	0.36333	− 1.371	0.1703	6.100000e-01	0.26	1.150000e+00
RLS-15 learning index	− 0.09413	0.04456	− 2.113	0.0346*	9.100000e-01	0.82	9.800000e-01
Incompatibility task (RT)	0.05987	0.02395	2.499	0.0124*	1.060000e+00	1.02	1.130000e+00
Incompatibility task (errors)	− 0.16328	0.16442	− 0.993	0.3207	8.500000e-01	NA	1.040000e+00
Literal fluency	− 0.19403	0.10392	− 1.867	0.0619	8.200000e-01	0.65	9.900000e-01

^*^Identifies significant differences with *p* < 0.05

^**^Identifies significant differences with *p* < 0.001

**Table 7:** Neuropsychological and demographic factors contributing to the presence of a dysexecutive syndrome in post-COVID-19 patient

Dysexecutive syndrome							
Coefficients							
	Estimate	Std. Error	z value	Pr(>|z|)	or	2.5%	97.5%
(Intercept)	42.86904	17.27180	2.482	0.01306*	4.147492e+18	4103792.70	8.00245e+36
MOCA score	− 1.41335	0.54850	− 2.577	0.00997**	2.400000e-01	0.06	5.90000e-01
Incompatibility task (RT)	0.12737	0.04455	2.859	0.00425**	1.140000e+00	1.06	1.27000e+00
Anxiety score (HARS)	− 0.32944	0.16824	− 1.958	0.05021	7.200000e-01	0.46	9.30000e-01
Physical QOL (SF-36)	− 0.02050	0.02712	− 0.756	0.44954	9.800000e-01	0.93	1.04000e+00
Mental QOL (SF-36)	− 0.13623	0.06158	− 2.212	0.02694*	8.700000e-01	0.75	9.60000e-01

^*^Identifies significant differences with *p* < 0.05

^**^Identifies significant differences with *p* < 0.001

With respect to cognitive complaints, the factors contributing to the presence of a cognitive complaint were the MOCA test score, the reaction time at the flexibility task (TAP), the fatigue scale score (Chalder), the score on the neuropathic pain scale (DN-4 scale), and the score on the dissociation scale (DES).

The presence of neuropsychological impairment at assessment seemed to be linked to the total score on the MOCA test, the learning index on the verbal episodic memory test (RLS-15), errors and reaction times on the interference management test (incompatibility task TAP), and literal fluency (number of correct responses).

The presence of a dysexecutive syndrome on neuropsychological assessment was related to the total score on the MOCA test, the response times on the interference management test (incompatibility task TAP), the score on the anxiety scale (HARS), and the mental and physical QOL scores (SF-36).

### Consequences of COVID-19 on professional activity

Data concerning the consequences of COVID-19 infection on the professional activity are presented in Table 8. A majority of the patients had an impact of COVID-19 on their professional activity (72%), with work stoppage in particular (68%). The median duration of work stoppage was 69.5 days [31–168.5]. In detail, 39% of patients were off work for 3 months, 11% for 3 to 6 months, and 15% for more than 6 months. There was no significant difference concerning the prescription of work stoppage and its duration, between the patients with or without cognitive disorders.

**Table 8:** Consequences of COVID-19 on the professional activity of the sample (*N *=74)

Work stoppage duration	Med (Q1-Q3)	P
	69.5 (31–168.5)	0.06
	*N *(%)	*P*
Impact of COVID-19 on professional activity	53 (72)	1
Yes		
No	14 (19)	
NA	7 (9)	
Work stoppage		0.81
Yes	50 (68)	
No	3 (4)	
NA	21 (28)	
Work stoppage duration		0.47
3 months	29 (39)	
3–6 months	8 (11)	
>6 months	11 (15) 26 (35)	
NA		

## Discussion

The aim of this study was to characterize the cognitive and psychological profiles of patients who presented with a cognitive complaint one year after hospitalization for severe COVID-19 infection through extended neuropsychological and psychological evaluations.

First, whereas all included patients presented a cognitive complaint measured at the QPC, approximately 27% of our sample presented normal performance at the extended neuropsychological evaluation. They therefore presented a contrast between a high level of complaints and an absence of cognitive disorders measured with neuropsychological tests. It seems possible, however, that more than these 27% patients did not have cognitive complaints following COVID-19 infection, if we take into account the fact that 57% of patients contacted did not wish to benefit from a full neuropsychological evaluation. Indeed, it is possible that at least some of these patients had no cognitive complaints and no neuropsychological post-COVID-19 repercussions, as it is possible that some of them did not want to take part, because they were not feeling well, or did not want to be involved in more hospital protocols at this particular time.

As described in the literature [6], cognitive complaints did not appear to be automatically correlated with the presence of cognitive disorders in post-COVID-19 patients. Interestingly, in our sample, the contributing factors to cognitive complaints were multidimensional, with neuropsychiatric (dissociation, neuropathic pain, and fatigue) and neurocognitive (general cognitive level and flexibility difficulties) dimensions. On the one hand, in some patients, cognitive complaints may be more closely related to other explanatory factors, such as the presence of psychiatric disorders (anxiety-depressive disorders, posttraumatic stress disorders,…), disabling fatigue, sleep disorders or pain [3, 4, 8], or even functional cognitive disorders [15]. We observed that, in our sample, even if no difference was observed when patients with and without cognitive disorders were compared, patients with cognitive disorders tended to present more frequently apathy, neuropathic pain or dissociative symptoms and more generally, a certain percentage of our sample presented anxiodepressive symptomatology, pathological fatigue, and drowsiness.

On the other hand, it is possible that a particularly high socioeducational level may have an impact on the interpretation of neuropsychological results, as the proposed tests may have restricted sensitivities in this specific case. The patients'performance was within the expected norms for their age and socioeducational level, but we were unable to compare their performance with that of their premorbid condition. This is all the more true given that, in the group without cognitive disorders, there is a high representation of high socioeducational levels compared with lower levels, and these patients tend to be more confronted with subtle changes in cognitive performance compared with their premorbid level, which hinders them in their daily and sometimes professional activities but cannot always be measured via psychometric tests. Even if the neuropsychological evaluation reveals no objectified disorders, the presence of a cognitive complaint calls for neurological and neuropsychological follow-up, on the one hand, and therapeutic education, on the other hand, to enable the patient to better understand the perceived subjective difficulties and find adjustments to cope with them. This is particularly important, as we observed that mental QOL is clearly impaired in patients with cognitive disorders, specifically those with dysexecutive syndrome.

Second, a majority of patients in our sample, i.e., 73%, presented with cognitive disorders, confirming the presence of post-COVID-19 neuropsychological disorders. Interestingly, the frequency and duration of work stoppages related to COVID-19 infection did not differ according to the presence or absence of cognitive impairment. To detect cognitive disorders associated with cognitive complaints, the use of the MOCA initially seems interesting, as it offers the possibility to discriminate patients with and without cognitive impairment and to distinguish patients with dysexecutive disorders from those with more diffuse cognitive profiles or with predominant memory profiles. Among these patients with neuropsychological abnormalities, the largest proportion were affiliated with the dysexecutive profile group, which is consistent with the literature [1, 5, 6, 10, 16, 17]. Indeed, although the ratio of single- versus multiple-domain impairments (i.e., affecting respectively one versus several cognitive domains) was reversed compared with that reported by Garcia-Sanchez [5] (single-domain 39,7%; multiple-domain 60,3%), since the sample contained more single- than multiple-domain profiles, the core of the disorders lies in executive functions. Whereas Garcia-Sanchez [5] identified attentional disorders as central to both types of profiles and executive impairment associated only with other impairments in the multiple-domain profile, the present cohort specifically identified executive disorders as transversal to both profiles. Studies seem to agree on the existence of different neuropsychological phenotypes [5, 6, 11, 14], which is certainly linked to the multifactorial nature of COVID-19 infection and its management [12].

These patients presented with dysexecutive syndrome, characterized by impaired performance in various processes, such as flexibility, interference resistance, updating in working memory, verbal initiation, and processing speed. These dysexecutive difficulties seem to impact retrieval in verbal episodic memory, specifically during the learning phase of the task. Indeed, executive functions imply strategic processes implicated in learning, as they are more generally implicated in the control of other cognitive processes [5]. They also presented particularly impaired mental QOL in comparison to patients with a memory profile or with no cognitive disorders. This could have led us to believe that dysexecutive disorders in this population have an impact on QOL due to the particular status of executive processes in general cognition and the major functional impact of dysexecutive disorders. However, these patients did not present other episodic memory or instrumental disorders. Regression analysis confirmed that the main factors contributing to the dysexecutive profile were general cognitive level, interference resistance, anxiety score, and QOL, which is consistent with that discussed previously.

Interestingly, as dysexecutive disorders appear through computerized tasks with response times as well as errors and omission records, a more ecological task for planning, i.e., the shopping task, was not impaired in these patients. Moreover, classical tasks for short-term and working memory, i.e., forward and backward spans, were not impaired. One hypothesis is that these tasks are nonspecific and may not be difficult enough to measure post-COVID-19 patients’ cognitive difficulties, particularly in adults still of working age. This highlights the necessity, for some of these patients with higher educational levels, to use selected tests for increased sensitivity to detect subtle dysexecutive disorders, as suggested by Daroische et al. [20] for neuropsychological diagnosis as well as rehabilitation management [12].

Third, it appears interesting to note that patients, regardless of whether they presented a cognitive impairment related to their initial cognitive complaints, did not present differences in manifestations of psychiatric disorders, pain, fatigue, or somnolence. Similarly, no differences between groups were observed in these dimensions. This population, with cognitive complaints and objectivized or non-objectivized neuropsychological disorders after severe COVID-19 infection, seemed to present, to some extent, a preserved psychiatric profile, whereas some of the patients presented anxiodepressive symptoms, a minority of whom had major depression. Patients with cognitive disorders tended to present more dissociative symptoms, apathy, and neuropathic pain, but this was not the case for the whole sample. This is relatively contradictory to many data in the literature [3, 4, 6, 8] and to our perception, as we wrote the present project. Indeed, the first patients who we took on during April 2020 in the neurological department presented with cognitive disorders, i.e., dysexecutive profiles associated with anxiodepressive symptomatology, and in some cases, psychotraumatic symptomatology associated with respiratory symptoms, fear of dying, and the highly anxiety-provoking context of this first wave of the epidemic in the country. We can thus postulate that cognitive complaints, especially in the case of a normal neuropsychological evaluation, cannot be explained solely by underlying psychiatric disorders, as suggested previously. Our study population is, however, very specific, with severe initial COVID-19 infection, older than the long COVID-19 population in which many specialized centers are currently in follow-up, with mostly young and female populations and nonsevere initial infection but important cognitive complaints.

Finally, consistent with the previous findings, the main factors contributing to cognitive disorders in post-COVID-19 patients were executive dysfunctions (verbal initiation and interference resistance), which are associated with general cognition, and learning ability in verbal episodic memory. The general profile of our sample with cognitive disorders seems to be quite similar to the “neurological” profile rather than the “psychiatric” profile described by Voruz et al. [6]. In fact, they were comparable in several respects, such as their age, educational level, and type of cognitive disorders observed, especially dysexecutive, memory, and language disorders.

These data are very important, as they confirm the persistence of cognitive complaints over time, 9 months after hospital discharge post-COVID-19 patients, and that cognitive disorders are frequent and predominantly based on dysexecutive disorders. However, these data also raise the question of the factor(s) inducing cognitive disorders in some but not all patients, particularly risk factors for specific medical antecedents and the severity of COVID-19 infection.

This work has several limitations and highlights. The limitations of the study include the size of the sample, particularly with low affiliation with certain subgroups. Although a high proportion of patients presented with cognitive complaints, many did not wish to undergo the proposed neuropsychological assessment and neurological follow-up. The lack of psychological measure during screening phase, as these factors may be involved in cognitive complaints. Perhaps, the 1-year follow-up initially proposed is too short, considering that we are now around 4 years after the start of the pandemic. The highlights of the study are its methodology, its prospective nature, and the presence of an extensive and specific cognitive and psychiatric assessment proposed one year after COVID-19 hospitalization in patients with cognitive complaints.

## Conclusion

Taking cognitive complaints into account is essential in the management of long-term COVID-19 syndrome. Neuropsychological evaluation could lead to the identification of objective cognitive disorders, particularly dysexecutive symptoms. In some cases, even in the case of normal cognitive performance, neuropsychological evaluation could help identify factors contributing to cognitive complaints and thus optimize the multidisciplinary management of patients.

## Appendix

### Measurements

*Cognitive assessment* targeting a younger patient population was considered extensive, with evaluation of general cognition, episodic memory, executive functions, instrumental functions, and autonomy. General cognition was measured with the MOCA scale [34], a 30-point composite scale with a total score < 26 indicating a possible cognitive deficit in adult patients.

Patient autonomy was evaluated with a 4-point IADL scale (Instrumental Activities of Daily Living; [52]), which assesses the ability to use a phone, to use means of transport, and to manage finances and medical treatments.

Episodic memory was evaluated in verbal and nonverbal conditions. Verbal episodic memory was assessed with the RLS-15 (35), a 15-item list that had to be learned over ten trials; the patients attempted to recall as many words as possible during each trial, and the neuropsychologist recalled only the words that had been forgotten from one trial to the next. The patient was required to recall the 15 words during a 20-min delay and, if not successfully, to recognize the 15 words among the distractors. Pathological performance was identified by comparing the patients’ scores to norms for age and educational level, for the mean recall (MR), the learning-consistency (LC) score, and the delayed-recall (DR) score.

Episodic nonverbal memory was assessed with the Rey–Osterrieth complex figure (ROCF; 36). The patient first had to copy the geometric figure without being informed that it constituted a memory test. The copy time and strategy were noted. Then, the patient had to recall the geometric figure after 3- and 30-min delays. A recognition task was proposed after delayed recall. Once again, performances were compared to norms for age and educational level.

Short-term memory was investigated with the verbal direct span from the WAIS-IV (37). Working memory was tested with the verbal indirect span from the WAIS-IV (37), and updating was tested with the working memory task of the TAP battery (38). The patient saw a number flashing up on the screen and had to answer as fast as possible when the item on the screen matched the one that appeared two items previously. The reaction time, errors, and omissions were recorded.

Concerning executive functions, processing speed was evaluated with the coding task from the WAIS-IV (37), in which the patient had to code as many items as possible. The flexibility task from the TAP battery (38) was proposed to test flexibility: the patient saw a letter and a number simultaneously and had to answer as fast as possible by alternating letters and numbers. The reaction time, errors, and omissions were recorded. Interference resistance was evaluated with the incompatibility task of the TAP battery (38): arrows appeared to the left or right of the screen, and the patient had to answer as fast as possible concerning the direction of the arrow (and not the side of the screen); reaction time, errors, and omissions were recorded. Item generation was tested by a verbal fluency task (39), in which patients had to generate as many words as possible starting with the letter “p” in 2 min; correct answers and errors were registered. Planning was evaluated by an ecological shopping task (40), in which patients had to organize the order of realization of 11 tasks, with geographical, time, duration, and weight constraints; the number of errors and duration of realization of the task were recorded.

Instrumental functions were evaluated for language, visual agnosia, constructive abilities, and apraxia. Concerning language, a denomination task (DO80 (41)) of 80 pictures was proposed, and the number of correct and incorrect answers was recorded. A phonological fluency task (39), in which as many names of animals as possible had to be given in 2 min by the patients, was proposed, and correct answers and errors were registered. Visual agnosia was evaluated with the help of the denomination task DO80 (41), and visuoconstructive abilities were evaluated with a copy of the Rey geometric complex figure. Apraxia was evaluated with the brief Mahieux-Laurent scale (42), which involves evaluating symbolic gestures, action mimes, and abstract gestures on 5, 10, and 8 points, respectively.

For the *psychological dimensions*, patients were assessed for depression by the Montgommery and Asberg Depression Rating Scale [MADRS (43)], with a cut-off score of 15 indicating depression; and ii. anxiety according to the Hamilton Anxiety Rating Scale [HARS (44)], with a score between 6 and 14 indicating minor anxiety and a score over 15 indicating major anxiety; and iii. apathy according to the Lille Apathy Rating Scale [LARS (45)], with scores indicating an absence of apathy (scores between − 36 and − 22), an apathy tendency (scores between -21 and − 17), moderate apathy (scores between − 16 and − 10), and severe apathy (scores between − 9 and + 36). Psychotrauma was evaluated with the Post-traumatic Stress Disorder Checklist for DSM-5 [PCL-5 (46)], a 20-item self-questionnaire with responses rated on a 5-point Likert scale (0 not at all, 4 extremely), with a total score of 80 points, with a score ≥ 33 indicating the presence of psychotrauma. Dissociation was evaluated with the Dissociative Experience Questionnaire [DES (47)], a 28-item self-rated questionnaire in which each item is rated between 0 and 100%, with a total score ≥ 25 indicating a greater probability of dissociative disorder.

*Additional questionnaires* have been proposed to evaluate QOL, pain, somnolence, and tiredness. QOL was evaluated with the 36-item Short Form Survey (SF-36, 48), which distinguishes physical and mental QOL, with dimensional scores between 0 and 100 and higher scores indicating better QOL. Pain was evaluated with a 10-point visual-analogical scale at different moments and situations during the previous 24 h, and neuropathic pain was evaluated with the DN4 scale (49), a 4-item scale with 10 items rated as yes or no, a total score of 10, and a score ≥ 4 indicating the presence of neuropathic pain. Tiredness was evaluated with the Chalder scale (50), an 11-item self-rated questionnaire; each item is rated from 0 to 3, and a total score > 11/33 indicates pathological tiredness. Somnolence was evaluated with the Epworth scale (51), an 8-item self-rated scale. Each item is rated from 0 to 3, corresponding to the probability of falling asleep in different situations. The total score is 24; a score between 9 and 14 corresponds to a lack of sleep, and a score > 15 corresponds to excessive somnolence.

## Mood, psychological dimensions, and quality of life

**Table 9:** Psychological, psychiatric, and quality-of-life scores for the total sample (N = 74) and for the subgroups of patients with and without cognitive disorders

Tests	Scores classification [n(%) or med(Q1, Q3)]	Total sample N = 74	Patients without cognitive disorder N = 20	Patients with cognitive disorders N = 54	P
MADRS	Mild depression	24 (32)	7 (35)	17 (31)	1
	Moderate depression	12 (16)	3 (15)	9 (17)	
	Severe depression	2 (3)	0 (0)	2 (4)	
	Normal score	36 (49)	10 (50)	26 (48)	
HARS	Major anxiety	29 (39)	5 (25	24 (44)	0.37
	Minor anxiety	29 (39)	10 (50)	19 (35)	
	Normal score	16 (22)	5 (25)	11 (20)	
LARS	Moderate apathy	4 (5)	0 (0)	4 (7)	0.39
	Severe apathy	5 (7)	0 (0)	5 (9)	
	No apathy	54 (73)	16 (80)	38 (70)	
	Tendency toward apathy	10 (14)	4 (20)	6 (11)	
	NA	1 (1)	0 (0)	1 (2)	
PCL-5	Normal	56 (76)	17 (85)	39 (72)	0.51
	PTSD	15 (20)	3 (15)	12 (22)	
	NA	3 (4)	0 (0)	3 (6)	
DES	Dissociation	9 (12)	1 (5)	8 (15)	0.21
	No dissociation	60 (81)	19 (95)	41 (76)	
	NA	5 (7)	0 (0)	5 (9)	
DN-4	Negative	44 (59)	16 (80)	28 (52)	0.07
	Positive	25 (34)	3 (15)	22 (41)	
	NA	5 (7)	1 (5)	4 (7)	
Chalder	Abnormal	48 (65)	13 (65)	35 (65)	1
	Normal	22 (30)	6 (30)	16 (30)	
	NA	4 (5)	1 (5)	3 (6)	
Epworth	No sleep deficit	37 (50)	11 (55)	26 (48)	0.77
	Sleep deficit	28 (38)	7 (35)	21 (39)	
	Signs of excessive somnolence	6 (8)	2 (10)	4 (7)	
	NA	3 (4)	0 (0)	3 (6)	
SF-36	Physical QOL	52.3 (32.16, 77.35)	67.81 (47.13, 80.78)	46.95 (25.47, 74.06)	0.07
	Mental QOL	61.35 (34.58, 77.42)	72.12 (49.89, 78.35)	47.83 (26.88, 75.25)	0.04*

*Identifies significant differences with p < 0.05

## References

[1] Ardila A, Lahiri D (2020) Executive dysfunction in COVID-19 patients. Diabetes Metab Syndr Clin Res Rev 14(5):1377–8 10.1016/j.dsx.2020.07.032 PMC7373676 32755837

[2] Zhou H, Lu S, Chen J, Wei N, Wang D, Lyu H et al (2020) The landscape of cognitive function in recovered COVID-19 patients. J Psychiatr Res 129:98–102 32912598 10.1016/j.jpsychires.2020.06.022 PMC7324344

[3] Cristillo V, Pilotto A, Piccinelli SC, Gipponi S, Leonardi M, Bezzi M et al (2022) Predictors of “brain fog” 1 year after COVID-19 disease. Neurol Sci 43(10):5795–5797 35930181 10.1007/s10072-022-06285-4 PMC9361921

[4] Rass V, Beer R, Schiefecker AJ, Lindner A, Kofler M, Ianosi BA et al (2022) Neurological outcomes 1 year after COVID-19 diagnosis: a prospective longitudinal cohort study. Euro J of Neurology 29(6):1685–1696 10.1111/ene.15307 PMC9111823 35239247

[5] García-Sánchez C, Calabria M, Grunden N, Pons C, Arroyo JA, Gómez-Anson B et al (2022) Neuropsychological deficits in patients with cognitive complaints after COVID-19. Brain and Behavior 12(3):e2508 35137561 10.1002/brb3.2508 PMC8933779

[6] Voruz P, Allali G, Benzakour L, Nuber-Champier A, Thomasson M, Jacot De Alcântara I et al (2022) Long COVID neuropsychological deficits after severe, moderate, or mild infection. CTN 6(2):9

[7] Costas-Carrera A, Sánchez-Rodríguez MM, Cañizares S et al (2022) Neuropsychological functioning in post-ICU patients after severe COVID-19 infection: the role of cognitive reserve. Brain Behav Immun Health 30(21):100425 10.1016/j.bbih.2022.100425 PMC8818554 35156065

[8] Dondaine T, Ruthmann F, Vuotto F, Carton L, Gelé P, Faure K et al (2022) Long-term cognitive impairments following COVID-19: a possible impact of hypoxia. J Neurol 269(8):3982–3989 35325308 10.1007/s00415-022-11077-z PMC8944178

[9] Priftis K, Velardo V, Vascello MGF, Villella S, Galeri S, Spada MS et al (2022) Limited evidence for neuropsychological dysfunction in patients initially affected by severe COVID-19. Neurol Sci 43(12):6661–6663 36050424 10.1007/s10072-022-06373-5 PMC9436465

[10] Benzakour L, Assal F, Péron JA (2021) Covid long neuropsychologique : origine neurologique ou psychiatrique ? Rev Med Suisse 17(736):822–826 33908718

[11] Voruz P, Jacot De Alcântara I, Nuber-Champier A, Cionca A, Allali G, Benzakour L et al (2023) Frequency of abnormally low neuropsychological scores in post-COVID-19 syndrome: the Geneva COVID-COG cohort. Arch Clin Neuropsychol 38(1):1–11 35942646 10.1093/arclin/acac068 PMC9384624

[12] Houben S, Bonnechère B (2022) The impact of COVID-19 infection on cognitive function and the implication for rehabilitation: a systematic review and meta-analysis. IJERPH 19(13):7748 35805406 10.3390/ijerph19137748 PMC9266128

[13] Ferrucci R, Dini M, Groppo E, Rosci C, Reitano MR, Bai F et al (2021) Long-lasting cognitive abnormalities after COVID-19. Brain Sci 11(2):235 33668456 10.3390/brainsci11020235 PMC7917789

[14] Voruz P, De Alcântara IJ, Nuber-Champier A, Cionca A, Guérin D, Allali G et al (2024) Persistence and emergence of new neuropsychological deficits following SARS-CoV-2 infection: a follow-up assessment of the Geneva COVID-COG cohort. J Glob Health 14:05008 38452292 10.7189/jogh.14.05008 PMC10919907

[15] Ball HA, McWhirter L, Ballard C, Bhome R, Blackburn DJ, Edwards MJ, Fleming SM, Fox NC, Howard R, Huntley J, Isaacs JD, Larner AJ, Nicholson TR, Pennington CM, Poole N, Price G, Price JP, Reuber M, Ritchie C, Rossor MN, Schott JM, Teodoro T, Venneri A, Stone J, Carson AJ (2020) Functional cognitive disorder : dementia’s blind spot. Brain 143:2895–2903 32791521 10.1093/brain/awaa224 PMC7586080

[16] Aiello EN, Radici A, Mora G, Pain D (2022) Cognitive phenotyping of post-infectious SARS-CoV-2 patients. Neurol Sci 43(8):4599–4604 35604618 10.1007/s10072-022-06130-8 PMC9125346

[17] Sadowski J, Klaudel T, Rombel-Bryzek A, Bułdak R (2024) Cognitive dysfunctions in the course of SARS-CoV-2 virus infection, including NeuroCOVID, frontal syndrome and cytokine storm (Review). Biomed Rep 21(1):103 38800038 10.3892/br.2024.1791 PMC11117100

[18] Mazza MG, Palladini M, De Lorenzo R, Magnaghi C, Poletti S, Furlan R et al (2021) Persistent psychopathology and neurocognitive impairment in COVID-19 survivors: effect of inflammatory biomarkers at three-month follow-up. Brain Behav Immun 94:138–147 33639239 10.1016/j.bbi.2021.02.021 PMC7903920

[19] Tarsitani L, Vassalini P, Koukopoulos A, Borrazzo C, Alessi F, Di Nicolantonio C et al (2021) Post-traumatic stress disorder among COVID-19 survivors at 3-month follow-up after hospital discharge. J Gen Intern Med 36(6):1702–1707 33782888 10.1007/s11606-021-06731-7 PMC8007055

[20] Daroische R, Hemminghyth MS, Eilertsen TH, Breitve MH, Chwiszczuk LJ (2021) Cognitive impairment after COVID-19—a review on objective test data. Front Neurol 12:699582 34393978 10.3389/fneur.2021.699582 PMC8357992

[21] Rogers JP, Chesney E, Oliver D, Pollak TA, McGuire P, Fusar-Poli P et al (2020) Psychiatric and neuropsychiatric presentations associated with severe coronavirus infections: a systematic review and meta-analysis with comparison to the COVID-19 pandemic. Lancet Psychiatry 7(7):611–627 32437679 10.1016/S2215-0366(20)30203-0 PMC7234781

[22] Kumar S, Veldhuis A, Malhotra T (2021) Neuropsychiatric and cognitive sequelae of COVID-19. Front Psychol 12:577529 33737894 10.3389/fpsyg.2021.577529 PMC7960660

[23] Pilotto A, Cristillo V, Cotti Piccinelli S, Zoppi N, Bonzi G, Sattin D et al (2021) Long-term neurological manifestations of COVID-19: prevalence and predictive factors. Neurol Sci 42(12):4903–4907 34523082 10.1007/s10072-021-05586-4 PMC8439956

[24] Van Helvoort MA, Pop-Purceleanu M, Tendolkar I, Everaerd DS (2021) Neuropsychiatric recovery after COVID-19—an observational cohort study. Tijdschr Voor Psychiatrie 63(7):514–521 34523701

[25] Pistarini C, Fiabane E, Houdayer E, Vassallo C, Manera MR, Alemanno F (2021) Cognitive and emotional disturbances due to COVID-19: an exploratory study in the rehabilitation setting. Front Neurol 12:643646 34079511 10.3389/fneur.2021.643646 PMC8165252

[26] Sánchez-García AM, Martínez-López P, Gómez-González AM, Rodriguez-Capitán J et al (2023) Post-intensive care unit multidisciplinary approach in patients with severe bilateral SARS-CoV-2 pneumonia. Int J Med Sci 20(1):1–10 36619225 10.7150/ijms.77792 PMC9812800

[27] Vialatte de Pémille C, Ray A, Michel A et al (2022) Prevalence and prospective evaluation of cognitive dysfunctions after SARS due to SARS-CoV-2 virus The COgnitiVID study. Rev Neurol (Paris) 178(8):802–807 35610098 10.1016/j.neurol.2022.03.014 PMC9123423

[28] Gouraud C, Bottemanne H, Lahlou-Laforêt K, Blanchard A, Günther S, Batti SE, Auclin E, Limosin F, Hulot JS, Lebeaux D, Lemogne C (2021) Association between psychological distress, cognitive complaints, and neuropsychological status after a severe COVID-19 episode: a cross-sectional study. Front Psych 12:725–861 10.3389/fpsyt.2021.725861 PMC8446522 34539470

[29] Cian V, De Laurenzis A, Siri C, Gusmeroli A, Canesi M (2022) Cognitive and neuropsychiatric features of COVID-19 patients after hospital dismission: an italian sample. Front Psychol 24(13):908363 10.3389/fpsyg.2022.908363 PMC9173000 35686079

[30] Ottonello M, Fiabane E, Nicolò Aiello E, Manera MR, Spada F, Pistarini C (2022) The association between objective cognitive measures and ecological-functional outcomes in COVID-19. Front Psychol 13:903697 36389563 10.3389/fpsyg.2022.903697 PMC9665234

[31] Ferrucci R, Dini M, Rosci C et al (2022) One-year cognitive follow-up of COVID-19 hospitalized patients. Eur J Neurol 29(7):2006–2014 35285122 10.1111/ene.15324 PMC9111730

[32] Birberg Thornberg U, Andersson A, Lindh M, Hellgren L, Divanoglou A, Levi R (2022) Neurocognitive deficits in COVID-19 patients five months after discharge from hospital. Neuropsychol Rehabil 33(10):1599–1623 36239662 10.1080/09602011.2022.2125020

[33] Thomas-Antérion C, Ribas C, Honoré-Masson S, Million J, Laurent B (2004) Evaluation de la plainte cognitive de patients Alzheimer, de sujets MCI, anxiodépressifs et de témoins avec le QPC (questionnaire de plainte cognitive). Neurol Psychiatr Gériatrie 4:30–34

[34] Nasreddine ZS, Phillips NA, Bédirian V, Charbonneau S, Whitehead V, Collin I et al (2005) The montreal cognitive assessment, MoCA: a brief screening tool for mild cognitive impairment. J Am Geriatr Soc 53(4):695–699 15817019 10.1111/j.1532-5415.2005.53221.x

[35] Rectem D, Poitrenaud J, Coyette F, Kalafat M, der Linden Van (2004) Une épreuve de rappel libre à 15 items avec remémoration sélective (RLS-15). In: Van der Liden M, Adam S, Agniel A, Baisset Mouly C et al (eds) L’évaluation des troubles de la mémoire: présentation de quatre tests de mémoire Épisodique (Avec Leur Étalonnage). Solal, Marseille, France

[36] Meyers JE, Meyers KR (1995) Rey complex figure test and recognition trial professional manual. Psychological Assessment Resources, Lutz FL USA

[37] Wechsler D (2008) Wechsler adult intelligence scale (WAIS–IV) Vol 22, 4th edn. NCS Pearson, San Antonio, TX, USA, p 1

[38] Zimmermann P, Fimm B (2007) Test for attentional performance (TAP), version 2.1 operating manual. PsyTest, Herzogenrath, Germany

[39] Godefroy O et le Groupe de Réflexion sur l'Evaluation des Fonctions EXécutives. (2008). Fonctions exécutives et pathologies neurologiques et psychiatriques**.** Marseille, Solal.

[40] Fournet N, Demazières-Pelletier Y, Favier S, Lemoine L. et Gros C. (2015). Test des commissions révisé. Dans L. Hugonot-Diener, C. Thomas-Antérion et F. Sellal (dir), Grémoire 2: tests et échelles des maladies neurologiques avec symptomatologie cognitive (p. 70–74). De Boeck-Solal.

[41] Deloche G, Hannequin D (1997) DO 80: Test de dénomination orale d’images. Paris éditeur Les Éditions du Centre de Psychologie Appliquée

[42] Mahieux-Laurent F, Fabre C, Galbrun E, Dubrulle A, Moroni C, Groupe de Réflexion Sur les Praxies du CMRR Ile-de-France Sud (2009) Validation of a brief screening scale evaluating praxic abilities for use in memory clinics evaluation in 419 controls, 127 mild cognitive impairment and 320 demented patients. Rev Neurol (Paris) 165(6–7):560–567 19150097 10.1016/j.neurol.2008.11.016

[43] Montgomery SA, Asberg M (1979) A new depression scale designed to be sensitive to change. Br J Psychiatry 1(134):382–389 10.1192/bjp.134.4.382 444788

[44] Hamilton M (1959) The assessment of anxiety states by rating. Br J Med Psychol 32(1):50–55 13638508 10.1111/j.2044-8341.1959.tb00467.x

[45] Sockeel P, Dujardin K, Devos D, Denève C, Destée A, Defebvre L (2006) The Lille apathy rating scale (LARS), a new instrument for detecting and quantifying apathy: validation in Parkinson’s disease. J Neurol Neurosurg Psychiatry 77(5):579–584 16614016 10.1136/jnnp.2005.075929 PMC2117430

[46] Ashbaugh AR, Houle-Johnson S, Herbert C, El-Hage W, Brunet A (2016) Psychometric validation of the English and French versions of the post-traumatic stress disorder checklist for DSM-5 (PCL-5). PLoS ONE 11:e0161645 27723815 10.1371/journal.pone.0161645 PMC5056703

[47] Bernstein EM, Putnam FW (1986) Development, reliability, and validity of a dissociation scale. J Nerv Ment Dis 174(12):727–735 3783140 10.1097/00005053-198612000-00004

[48] Ware JE, Sherbourne CD (1992) The MOS 36-item short-form health survey (SF-36). I. conceptual framework and item selection. Med Care 30(6):473–83 1593914

[49] Spallone V, Morganti R, D’Amato C, Greco C, Cacciotti L, Marfia GA (2012) Validation of DN4 as a screening tool for neuropathic pain in painful diabetic polyneuropathy. Diabet Med 29(5):578–585 22023377 10.1111/j.1464-5491.2011.03500.x

[50] Chalder T, Berelowitz G, Pawlikowska T, Watts L, Wessely S, Wright D et al (1993) Development of a fatigue scale. J Psychosom Res 37(2):147–153 8463991 10.1016/0022-3999(93)90081-p

[51] Johns MW (1991) A new method for measuring daytime sleepiness: the Epworth sleepiness scale. Sleep 14(6):540–545 1798888 10.1093/sleep/14.6.540

[52] Lawton MP, Brody EM (1969) Assessment of older people: self-maintaining and instrumental activities of daily living. Gerontologist 9(3, Pt 1):179–86 5349366

